# Optical coherence tomography evaluation of pulmonary arterial vasculopathy in Systemic Sclerosis

**DOI:** 10.1038/srep43304

**Published:** 2017-02-24

**Authors:** Johannes P. Schwaiger, Christopher D. Loder, David Dobarro, Thomas Kaier, Sally Reddecliffe, Benjamin E. Schreiber, Clive Handler, Christopher P. Denton, John G. Coghlan

**Affiliations:** 1 Department of Cardiology, Royal Free London NHS Foundation Trust, Pond Street, London, NW3 2QG, United Kingdom; 2 Department of Rheumatology. Royal Free London NHS Foundation Trust, Pond Street, London, NW3 2QG, United Kingdom

## Abstract

Our current understanding of the pathophysiology of pulmonary vascular disease is incomplete, since information about alterations of the pulmonary vasculature in pulmonary arterial hypertension (PAH) is primarily provided by autopsy or tissue specimens. The aim of this study was to compare the distal pulmonary vasculature of <2 mm in diameter in Systemic Sclerosis (SSc) patients with (n = 17) and without (n = 5) associated PAH using Optical Coherence Tomography during Right Heart catheterization. SSc-PAH patients showed significant thickening of Intima Media Thickening Area compared to patients without PAH (27 +/− 5.8% vs. 21 +/− 1.4%, p = 0.024). A good haemodynamic response to previous targeted PAH treatment was associated with a significantly greater number of small pulmonary artery side branches <300 μm per cm vessel (3.8 +/− 1.1 vs. 1.8 +/− 1.1; p = 0.010) and not associated with Intima Media thickening Area (26 +/− 5.4% vs. 28 +/− 6.7%; p = 0.6). Unexpected evidence of pulmonary artery thrombus formation was found in 19% of SSc-PAH patients. This is the first *in-vivo* study demonstrating a direct link between a structural abnormality of pulmonary arteries and a response to targeted treatment in PAH. Intravascular imaging may identify subgroups that may benefit from anticoagulation.

Our current understanding of the pathophysiology of pulmonary vascular disease is incomplete, since information about alterations of the pulmonary vasculature in pulmonary arterial hypertension (PAH) is primarily provided by autopsy or tissue specimens. Classical histological findings in idiopathic pulmonary arterial hypertension (IPAH) include medial and intimal hypertrophy as well as more distinctive changes in later stages, namely concentric laminar intimal fibrosis, plexogenic arteriopathy, fibrinoid necrosis and microthrombotic lesions^1^,^2^,^3^,^4^,^5^. In PAH associated with Systemic Sclerosis (SSc) however data point to a more distinct phenotype. Both historical^6^ and more recent studies suggest that SSc-PAH is characterised by significant intimal thickening, small vessel intimal fibrosis, and less plexiform arteriopathy with reports of a concomitant Pulmonary veno-occlusive disease pattern in some cases^7^,^8^.

Only few data are available about intravascular imaging of the pulmonary vasculature primarily due to the lack of adequate imaging techniques. However, in recent years a novel intravascular imaging modality has evolved, namely Optical coherence tomography (OCT), which is licensed for use in the coronary arteries. OCT is an imaging modality analogous to ultrasound using light instead of ultrasound and cross-sectional images are generated using the time delay and intensity of reflected light (backscatter) from internal tissues^9^,^10^,^11^.

Intravascular OCT is performed using a probe containing a single rotating optical fibre. The fibre can be delivered to the distal vessel using an over the wire delivery balloon, and the vessel either occluded or filled with saline from a proximal catheter during imaging.

OCT has successfully been used in several pulmonary hypertension (PH) populations with promising results. Dai *et al*. examined a large mixed PH cohort and found wall thickness to be significantly correlated with mean pulmonary artery pressure and in a small PAH subpopulation remodelling was observed after treatment^12^. Another group has studied a mixed PAH population and found wall thickness being predictive of outcome^13^. Also, a PH population with mitral valve disease was recently studied^14^.

This is the first study using OCT to systematically quantify *in-vivo* vessel wall thickness in small pulmonary arteries of less than 2 mm and the sub 300 μm side branches of these vessels that are the site of the main pathology in PAH. We then correlated morphological findings with haemodynamic parameters and response to targeted treatment.

## Results

17 patients met criteria for PAH with a mean pulmonary artery pressure (mPAP) of 39 +/− 12 mmHg and a Pulmonary Vascular Resistance (PVR) of 5.8 +/− 2.3 Wood Units (WU). In 16 patients OCT was performed during clinically indicated follow-up right heart catheterization (RHC), the mean period between initial diagnostic catheterization and follow-up RHC was 34 +/− 37 months (2–126 months; median 27 months). Mean pulmonary artery pressure (mPAP) at the time of diagnosis was 45 +/− 11 mmHg, mean PVR of 8.7 +/− 4.2 WU. In one patient OCT was performed during the initial diagnostic evaluation with first RHC. 5 patients with SSc had no evidence for PAH on RHC and formed a control group (mPAP 18 +/− 1 mmHg (Table 1).

We obtained 100 pull-backs in total, a mean of 4.5 per patient. 44% were performed in the Right Lower Lobe, 9% in the Right Upper Lobe, 47% in the Left Lower Lobe. 44 pull-backs were not analysed due to vessel size >2 mm (n = 25) and artefacts (n = 19). We were able to image at least one pulmonary artery of 2 mm or less in diameter in 21 out of 22 patients.

**Figure 1:**
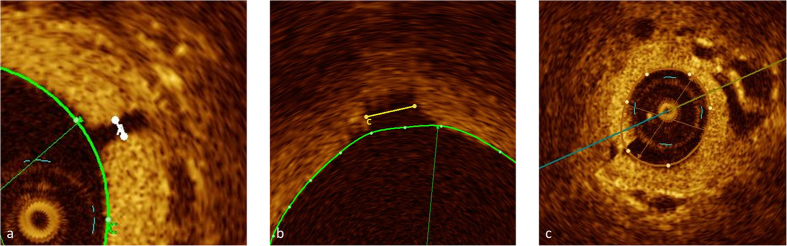
Small pulmonary artery side branches and the smallest pulmonary artery imaged. Coloured tracings represent endoluminal contours. (**a**) 60 μm side branch (measurement “A”) (**b**) 330 μm side branch (measurement “C”) (**c**) diameter of vessel approaching diameter of the OCT catheter (470 μm). A small stitch artefact at 7 o’ clock.

In 16 patients (12 PAH, 4 controls) at least one high quality pull-back without relevant artefacts (at least one uninterrupted segment of 10 mm in length) was available in a pulmonary artery of <2 mm in diameter. The smallest side branches identified were between 60 μm and 100 μm and the smallest pulmonary artery imaged approached the size of the OCT catheter (470 μm) (Fig. 1).

All the common artefacts known with OCT systems were observed^9^: most commonly poor displacement of blood, eccentric image wire leading to distortion artefacts, flow artefacts, sew-up artefacts, and difficulties with calibration due to wire fracture or small intraluminal foreign bodies, presumably dislocation of small bits of intima.

### Comparison SSc-PAH versus SSc without PAH

**Figure 2:**
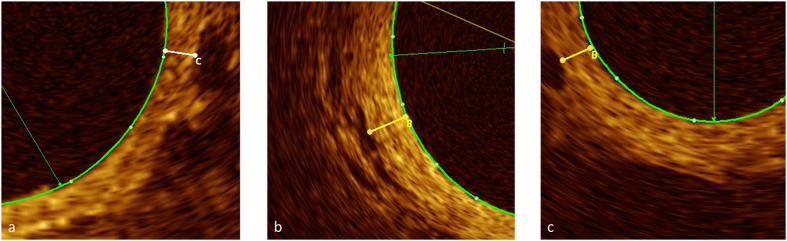
Examples of IMT measurement in patients with and without PAH. Clearly defined structure at outer border. All three pictures demonstrate eccentric IMT thickening with difficult delineation towards adventitia. Coloured tracings represent endoluminal contours. (**a**) SSc, no PAH. IMT 0.10 mm (measurement “C”). PA mean 19 mmHg (**b**) SSc-PAH. IMT 0.27 mm (measurement “B”). mPAP 61 mmHg (**c**) SSc-PAH. IMT 0.15 mm (measurement “B”). mPAP 46 mmHg.

**Figure 3:**
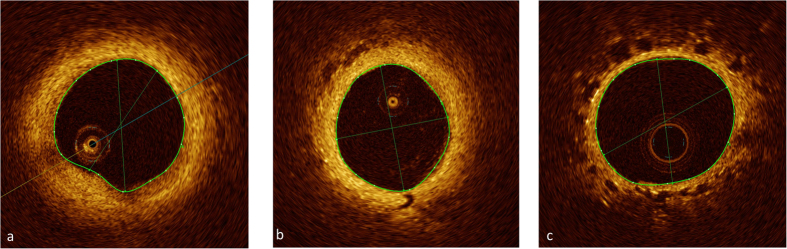
Examples of pulmonary arteries in patients with PAH (limited and good haemodynamic response) and without PAH. Coloured tracings represent endoluminal contours. (**a**) SSc-PAH. mPAP 51 mmHg. Treatment with Sildenafil. Limited haemodynamic response, 8% reduction in PVR. 0.2 side branches <300 μm per cm vessel (**b**) SSc-PAH. mPAP 29 mm Hg. Treatment with Bosentan. Good haemodynamic response, 51% reduction in PVR. 6.4 side branches <300 μm per cm vessel (**c**) SSc. mPAP 19 mmHg. 5.8 side branches <300 μm per cm vessel.

SSc-PAH patients showed significant thickening of the Intima Media Thickening Area (IMTA) at 2 mm diameter vessels (27 +/− 5.8% vs. 21 +/− 1.4%, p = 0.024) compared to SSc-patients without PAH (Table 2). Figs 2 and 3 demonstrate examples of pulmonary arteries in patients with and without PAH. The IMTA raw data set can be viewed online (Supplementary data S1).

**Figure 4:**
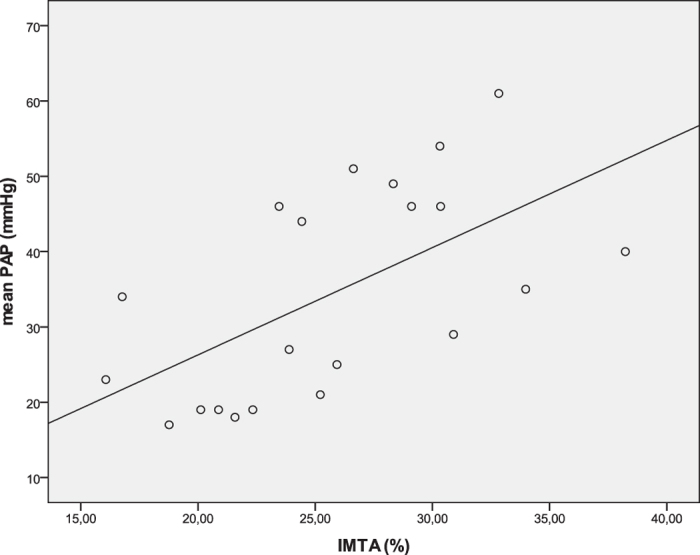
Graphical representation of the Pearson’s correlation between IMTA at 2 mm vessel level and mPAP at time of OCT (r = 0.60, p = 0.004).

In PAH a trend toward fewer side branches <300 μm was noted (2.6 +/− 1.5 vs. 4.3 +/− 1.9, p = 0.08) (Table 2). IMTA was significantly correlated with mPAP (r = 0.60, p = 0.004) (Fig. 4), but no correlation was found between mPAP and number of side branches <300 μm (r = −0.28; p = 0.29).

### Comparison good versus limited haemodynamic response in SSc-PAH

Patients with good haemodynamic response (GHR) and limited haemodynamic response (LHR) had similar haemodynamics at the time of OCT (right atrial pressure 6 +/− 3 vs. 8 +/− 1 mmHg; mPAP 37 +/− 13 vs. 39 +/− 10 mmHg; pulmonary artery wedge pressure 10 +/− 3 vs. 11 +/− 1 mmHg; Cardiac output 5.1 +/− 1.2 vs. 5 +/− 1.2l), but showed a more pronounced mean improvement of their NT-proBNP levels (330 vs. 94 pmol/l improvement; (NT-proBNP data in two patients in the GHR group not available)) and 6MWT results (293 m vs. 49 m improvement). Mean time on treatment was 45 months (range 2.1–126 months) in the GHR group and 27 months in the LHR group (range 3.2–61 months).

SSc-PAH patients with a GHR to targeted treatment had significantly greater number of side branches <300 μm per cm vessel when compared to patients with LHR (3.8 +/− 1.1 vs. 1.8 +/− 1.1; p = 0.010). When comparing patients with GHR to patients without SSc-PAH (controls) a very similar number of side branches <300 μm was found with no statistical difference (3.8 +/− 1.1 vs. 4.3 +/− 1.9; p = 0.6). IMTA was not statistical different when comparing GHR to LHR patients (26 +/− 5.4% vs. 28 +/− 6.7%; p = 0.6) (Table 2).

### Intravascular thrombus and webs

**Figure 5:**
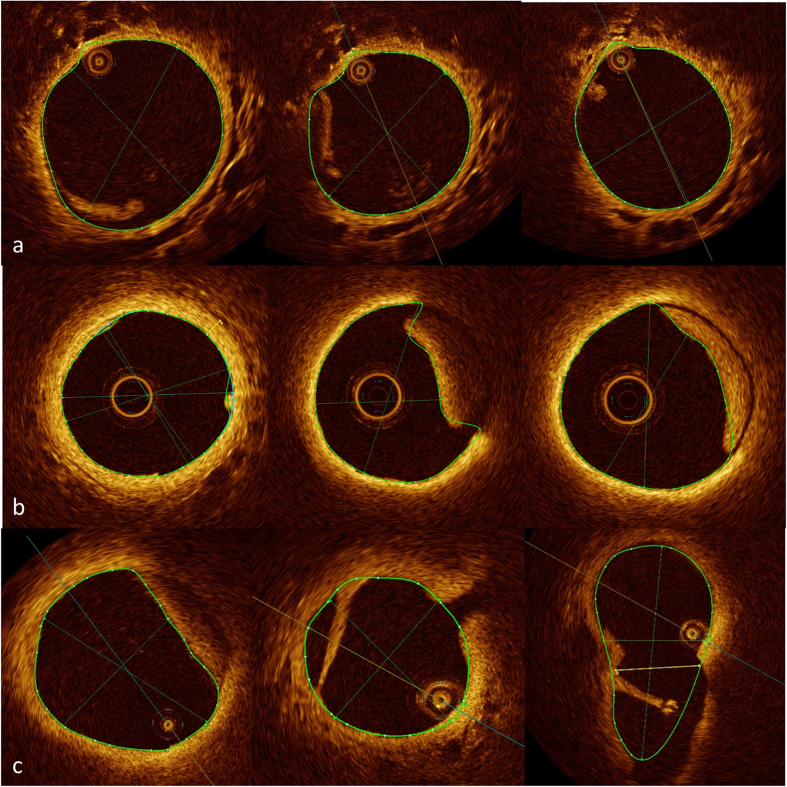
Intravascular thrombus formation in patients with SSc-PAH. Coloured tracings represent endoluminal contours. (**a**,**b**) SSc-PAH; wall-adherent thrombus (**c**) SSc-PAH; wall-adherent thrombus and web.

In 4 (of 16; 25%) SSc-PAH patients OCT revealed evidence of intravascular thrombus formation, however in 3 patients (19%) this was not known (negative previous Ventilation/Perfusion scan) (Fig. 5). In one patient (75, female, limited cutaneous SSc) thrombus formation was visible in a pulmonary artery in the right lower lobe, but this was known from a previous pulmonary angiogram showing modest, localised thromboembolic changes in the right middle and right lower lobe. In this patient Ventilation/Perfusion scanning was non-diagnostic. No thrombus was found in the SSc-patients without PAH.

## Discussion

This is the first study demonstrating the feasibility of routine evaluation of the distal pulmonary vasculature <2 mm in diameter using OCT in patients with PAH. In a Systemic Sclerosis population we have confirmed the secondary effects of pulmonary hypertension on larger vessel wall thickness demonstrated in other studies. In addition we found that the number of pulmonary artery side branches <300 μm from distal pulmonary arteries varies and may be of therapeutic significance and that distal thromboembolic abnormalities may be present despite negative non- invasive testing including Ventilation/Perfusion scanning.

As in previous OCT imaging studies we found that PH per se is associated with a significant thickening of pulmonary arteries^13^,^15^,^16^. Due to the novelty of OCT imaging in pulmonary arteries there is a lack of standardization as yet^17^. Previous OCT studies reported on more proximal vessels, usually 3–4 mm in diameter, where changes observed are likely to reflect secondary consequences of elevated pressures rather than the site of underlying pathology in the muscular pulmonary arterioles. Secondly these studies were performed in mixed PAH populations presenting the findings of diseases with varying aetiologies, natural histories and potentially different morphology. To avoid these problems we have only performed measurements at a pulmonary artery diameter of 2 mm and also focused on one PAH subgroup, namely on patients with SSc.

We were able to demonstrate for the first time a direct link between a structural abnormality of pulmonary arteries and a response to targeted treatment *in vivo*. Patients who previously responded well to targeted treatment (PVR reduction of >30%) had a greater number of very small pulmonary artery side branches (<300 μm in diameter) compared to patients who had a limited response to targeted treatment.

Our finding of the variation of the very small pulmonary vasculature is consistent with several previous studies, both historical and more recently. Medial hypertrophy and intimal proliferation leading to obliteration of small pulmonary arteries was already described in the 1970ies in patients that we would currently identify as having PAH^4^, more recently severely stenosed or occluded small pulmonary artery vessels were also seen in a 3D electron microscopy study^18^. In SSc-PAH previous autopsy studies have found significant intimal thickening in pulmonary arteries of all sizes on histology, with a high prevalence of luminal occlusion particularly in PAH associated with limited cutaneous SSc^6^. More recently, in a study in SSc patients with and without PAH some degree of intimal fibrosis was found in the arterial tree at all levels in histology specimens, and in SSc-PAH patients intimal fibrosis and/or obliteration was present throughout in small vessels at alveolar levels^7^.

Our finding of a reduced number of very small pulmonary artery side branches could be the direct consequence of this obliterative process and the first study demonstrating this phenomenon *in-vivo*. We cannot completely exclude the possibility of periprocedural vasoconstriction in susceptible patients, however vasoreactivity is known to be very rare in PAH associated with Systemic Sclerosis.

Our finding of the relation between number of side branches <300 μm and a limited haemodynamic response to targeted treatment is also consistent with a histology study done in “primary pulmonary hypertension” by Palevsky *et al*^3^. Here IMTA of >18% in vessels between 50 and 500 μm in a lung biopsy had an 85% predictive value for identifying patients with poor outcome.

In line with others we have found that OCT may be helpful in defining a thromboembolic component of pulmonary hypertension and therefore in determining the need for systemic anticoagulation in populations with an elevated risk of bleeding. It has long been appreciated that thrombotic or thromboembolic lesions are not uncommon in patients with pulmonary hypertension^4^ and patients with SSc-PAH^7^, in our study we found evidence for localized thrombus and webs in a subgroup of patients with SSc-PAH, which was not found on routine testing. The finding of *in situ* thrombosis and re-canalised webs in patients with negative Ventilation/Perfusion scans may explain the reported benefit of anticoagulation in most PAH studies. The negative findings in the COMPERA registry (Comparative, Prospective Registry of Newly Initiated Therapies for Pulmonary Hypertension)^19^ in patients with SSc may reflect the increased risk associated with anticoagulation, where this is not targeted at the subpopulation where benefit can be accrued. Our findings suggest that the conflicting data published in respect of the role of anticoagulation may be more nuanced than appreciated to date and may be relevant to the ongoing debate about the role of anticoagulation in this population.

OCT is the only technique to provide an *in vivo* insight into the morphological changes associated with PAH at the level of the vascular pathology. Current developments in the field of OCT may allow for even higher resolution (<5 μm)^20^, and combination with ultrasound technology may allow for more detailed exploration of the side-branch pathology^21^.

Our study has several limitations. One is the small number of patients, however robustness of the data presented has been increased by ensuring homogeneity in the study population – limiting the study to SSc-PAH patients where potential confounders (any left heart abnormality, any significant lung pathology and any renal involvement) have been excluded. Secondly this study does not answer the question whether responders to targeted treatment simply have preservation of small vessels available for vasodilatation or whether they have the potential to grow new vessels in response to therapy, longitudinal studies are needed. One small longitudinal OCT study is available which reported on pulmonary artery remodelling after treatment in early stage of PAH, however only wall thickening was assessed in more proximal vessels in the 3–4 mm range^12^.

We did not use computerized algorithm methods to measure thickening of the vessel walls for two reasons. We have encountered significant eccentric and patchy thickening^5^ which could only be overcome by complex algorithms over predefined pulmonary artery segments to clearly define the degree of thickening. In addition the outer boundary towards the adventitia is often difficult to delineate, in particular in PAH which makes automatic measurements error-prone. We have therefore relied on manual measurements accepting a certain degree of underestimation but making overestimation very unlikely.

In summary in this pilot study we have systematically assessed the morphology of small pulmonary arteries <2 mm in diameter using Optical Coherence Tomography and obtained information about the very small pulmonary vasculature <300 μm which plays a major role in resistance generation. We have found that in SSc-PAH the presence of side branches <300 μm arising from pulmonary arteries of <2 mm in diameter was associated with good haemodynamic response to targeted treatment. Furthermore OCT imaging revealed evidence for thrombosis not evident on previous Ventilation/Perfusion scanning in a subgroup of patients. This might contribute to unravelling the conflicting data published so far with regards to the benefit of anticoagulation in SSc-PAH.

## Methods

### *S*tudy subjects

SSc-patients who underwent diagnostic RHC were included for OCT of the pulmonary arteries at the Royal Free Hospital. Inclusion criteria required definite diagnosis of systemic sclerosis or a scleroderma spectrum connective tissue disease according to EULAR/ACR 2013 classification criteria. The indication for RHC was either diagnosis of PH or management of known PH. Patients had to be haemodynamically compensated at the time of procedure.

Exclusion criteria were pregnancy, age <18 or >80 years, allergy to heparin or contrast media, estimated glomerular filtration rate <45 ml/min, tortuous vascular anatomy limiting catheter manipulation, severe pulmonary fibrosis, musculoskeletal conditions which preclude prolonged recumbence and a borderline elevation of mean pulmonary artery pressure (21–24 mmHg) if the patient underwent initial diagnostic RHC.

### Good and limited haemodynamic response

SSc-PAH patients who reduced their PVR (at time of study RHC) by more than 30% compared to initial catheterization (prior to treatment initiation with targeted therapies) were classified as having a good haemodynamic response (GHR), whereas all other patients were classified as having limited haemodynamic response (LHR). The 30% threshold was chosen for the following reasons. In one longitudinal clinical and histology study a reduction in PVR of 30% was defined as vasodilatation^3^, but also more recently in a subgroup of the SERAPHIN study Macitentan, an endothelin receptor antagonist improving morbidity and mortality, reduced pulmonary vascular resistance by 25%^22^.

The study complies with the declaration of Helsinki and had been approved by the institutional ethics committee of the Royal Free Hospital in London. A written informed consent had been obtained from every patient. Baseline characteristics and haemodynamic data are given in Table 2.

### Optical coherence tomography

**Figure 6:**
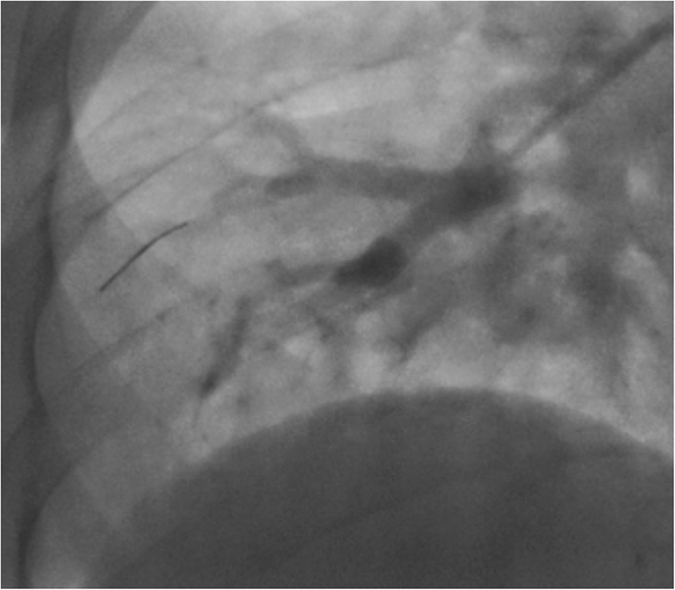
Swan-Ganz-Catheter in wedge position with selective pulmonary angiography in lower right lobe. OCT catheter in distal pulmonary artery.

Right heart catheterization was performed in a supine position via the femoral vein according to local and national guidelines. After the routine RHC procedure a LightLab^®^ 200 cm Cardiovascular imaging wire (LightLab Imaging Inc. UK; London) with an outer diameter of 470 μm was delivered via a 6 French balloon tipped catheter (“Arrow”; Teleflex Inc.) to a pulmonary artery mostly in the lower lobes with the goal of reaching a vessel of <2 mm in diameter (Fig. 6). After delivery of the OCT catheter via the Swan Ganz catheter the balloon was inflated and the distal vessel filled with a 50/50 contrast saline mixture during pull-back. Images were acquired at a pull-back speed of 1 mm/s. Pullbacks were then reviewed offline on dedicated St. Jude Software. All measurements were done by the first author.

### Intima media thickening area

Intima Media thickness (IMT) was measured at least twice in each patient (mean of 2.3 measurements per patient) at 2 mm vessel diameter (+/−10%). Significant patchy and eccentric thickening of IMT was very common (Figs 1, 2, 3) therefore all available pull-backs were rigorously examined for the site of the least wall thickness that was clearly visible, mostly due to adjacent vasa vasorum or alveolar tissues, similar to Li N^23^ (Fig. 1). Only high quality images without artefacts or significant distortion were used.

Similar to others we then calculated a hypothetic area of the vessel occupied by the IMT in percent of total vessel area, the IMTA, not taking into account the potential eccentric shape^3^,^24^. The vessel lumen was calculated using the mean vessel diameter as provided by the Imaging Software. The lumen of the vessel including IMT was then calculated using standard circle calculation formulas (A = r^2^π; r = (mean internal vessel diameter + 2*IMT)/2). IMTA is then presented as percent thickening of the vessel including Intimal Medial Thickening.

### Side branches

Uninterrupted (at least 10 mm in length) pulmonary artery segments <2 mm in diameter were screened for side branches <300 μm in diameter (Fig. 2). Results are presented as the number of side branches per cm vessel.

### Statistics

Statistical analysis was performed using SPSS 21.0. Normally distributed continuous data were expressed as mean and standard deviation. NT-proBNP levels were non-normally distributed and were reported as mean, median and interquartile range. Univariate comparisons were performed using unpaired t-tests. Relationships between variables were assessed using Pearson’s correlation coefficient. P-values < 0.05 were considered statistically significant.

## Additional Information

**How to cite this article**: Schwaiger, J. P. *et al*. Optical coherence tomography evaluation of pulmonary arterial vasculopathy in Systemic Sclerosis. *Sci. Rep.* **7**, 43304; doi: 10.1038/srep43304 (2017).

**Publisher's note:** Springer Nature remains neutral with regard to jurisdictional claims in published maps and institutional affiliations.

## Supplementary Material

Supplementary Information

## Figures and Tables

**Table 1:** baseline characteristics.

	PAH (n = 17)	Non-PAH (n = 5)
Age, *years*	62 +/− 12	62 +/− 5
Sex, *females*	13	4
LcSSC	15	5
NT-pro BNP, *pmol/l Mean; Median; IQR*	127, 30, 119	17, 15, 13
mPAP *(mmHg)*; PVR *(WU)*	39 +/− 12; 5.8 +/− 2.3	18 +/− 1; 1.7 +/− 0.4
RAP; PAWP (mmHg)	7.1 +/− 3.5; 9.7+/− 2.4	5 +/− 1.4; 10.5 +/− 0.9
CO (l/min)	5 +/− 1	4.7 +/− 1.4
Targeted treatment *Single/double/no therapy*	11/5/1*	n.a.
Time from onset of SSC *months*	128 +/− 83	130 +/− 102

CO (cardiac output), LcSSc (limited cutaneous systemic sclerosis), mPAP (mean pulmonary artery pressure), mPVR (mean pulmonary vascular resistance, PAH (pulmonary arterial hypertension), PAWP (pulmonary artery wedge pressure), RAP (right atrial pressure), SSc (systemic sclerosis), WU (wood unit).

*Among 11 patients with single targeted therapy 7 patients received Phosphodiesterase-5 inhibitors and 4 patients received Endothelin receptor antagonists. The remaining 5 patients were on combination therapy with a Phosphodiesterase-5 inhibitor and an Endothelin receptor antagonist.

**Table 2:** OCT measurements in patient groups.

	PAH n = 16 *n = 12	Non-PAH n = 5 **n = 4	p- value	GHR n = 7 *n = 5	LHR n = 8 **n = 7	p- value
IMTA 2 mm	27 +/− 5.8%	21 +/− 1.4%	**0.024**	26 +/− 5.4%	28 +/− 6.7%	0.6
SB/cm < 300 μm	2.6 +/− 1.5*	4.3 +/− 1.9**	0.08	3.8 +/− 1.1*	1.8 +/− 1.1**	**0.010**

GHR (good haemodynamic response), IMTA (Intima media thickness area), LHR (limited haemodynamic response), PAH (pulmonary arterial hypertension), SB (side branches).

## References

[b1] Edwards W. D. & Edwards J. E. Clinical primary pulmonary hypertension: three pathologic types. Circulation 56, 884–888 (1977). 912851 10.1161/01.cir.56.5.884

[b2] Mooi W. J. Pathology of pulmonary hypertension. Transplant. Proc. 19, 4373–4374 (1987). 3672623

[b3] Palevsky H. I. . Primary pulmonary hypertension. Vascular structure, morphometry, and responsiveness to vasodilator agents. Circulation 80, 1207–1221 (1989). 2805259 10.1161/01.cir.80.5.1207

[b4] Wagenvoort C. A. & Wagenvoort N. O. E. K. Primary Pulmonary Hypertension: A Pathologic Study of the Lung Vessels in 156 Clinically Diagnosed Cases. Circulation 42, 1163–1184 (1970).

[b5] Pietra G. G. . Pathologic assessment of vasculopathies in pulmonary hypertension. J. Am. Coll. Cardiol. 43, 25S–32S (2004). 15194175 10.1016/j.jacc.2004.02.033

[b6] Al-Sabbagh M. R. . Pulmonary arterial histology and morphometry in systemic sclerosis: a case-control autopsy study. J. Rheumatol. 16, 1038–1042 (1989). 2585400

[b7] Overbeek M. J. . Pulmonary arterial hypertension in limited cutaneous systemic sclerosis: a distinctive vasculopathy. Eur. Respir. J. 34, 371–379 (2009). 19282344 10.1183/09031936.00106008

[b8] Dorfmuller P. . Fibrous remodeling of the pulmonary venous system in pulmonary arterial hypertension associated with connective tissue diseases. Hum. Pathol. 38, 893–902 (2007). 17376507 10.1016/j.humpath.2006.11.022

[b9] Bezerra H. G., Costa M. A., Guagliumi G., Rollins A. M. & Simon D. I. Intracoronary optical coherence tomography: a comprehensive review clinical and research applications. JACC. Cardiovasc. Interv. 2, 1035–1046 (2009). 19926041 10.1016/j.jcin.2009.06.019 PMC4113036

[b10] Prati F. . Expert review document on methodology, terminology, and clinical applications of optical coherence tomography: physical principles, methodology of image acquisition, and clinical application for assessment of coronary arteries and atherosclerosis. Eur. Heart J. 31, 401–415 (2010). 19892716 10.1093/eurheartj/ehp433

[b11] Yabushita H. . Characterization of human atherosclerosis by optical coherence tomography. Circulation 106, 1640–1645 (2002). 12270856 10.1161/01.cir.0000029927.92825.f6

[b12] Dai Z. . OCT imaging for the management of pulmonary hypertension. JACC. Cardiovasc. Imaging 7, 843–845 (2014). 25124017 10.1016/j.jcmg.2014.01.020

[b13] Domingo E. . *In vivo* assessment of pulmonary arterial wall fibrosis by intravascular optical coherence tomography in pulmonary arterial hypertension: a new prognostic marker of adverse clinical follow-up. Open. Respir. Med. J. 7, 26–32 (2013). 23730366 10.2174/1874306401307010026 PMC3636492

[b14] Jorge E. . Pulmonary vascular remodeling in mitral valve disease: An optical coherence tomography study. Int. J. Cardiol. 203, 576–578 (2016). 26580335 10.1016/j.ijcard.2015.10.212

[b15] Hou J. . Pulmonary vascular changes in pulmonary hypertension: optical coherence tomography findings. Circ. Cardiovasc. Imaging 3, 344–345 (2010). 20484114 10.1161/CIRCIMAGING.109.882498

[b16] Tatebe S. . Optical coherence tomography as a novel diagnostic tool for distal type chronic thromboembolic pulmonary hypertension. Circ. J. 74, 1742–1744 (2010). 20501955 10.1253/circj.cj-10-0160

[b17] Jorge E. . Optical coherence tomography of the pulmonary arteries: A systematic review. J. Cardiol. 67, 6–14 (2016). 26572955 10.1016/j.jjcc.2015.09.024

[b18] Miura A. . Three-dimensional structure of pulmonary capillary vessels in patients with pulmonary hypertension. Circulation 121, 2151–2153 (2010). 20479166 10.1161/CIR.0b013e3181e037c1

[b19] Olsson K. M. . Anticoagulation and survival in pulmonary arterial hypertension: results from the Comparative, Prospective Registry of Newly Initiated Therapies for Pulmonary Hypertension (COMPERA). Circulation 129, 57–65 (2014). 24081973 10.1161/CIRCULATIONAHA.113.004526

[b20] Yonetsu T., Bouma B. E., Kato K., Fujimoto J. G. & Jang I. K. Optical coherence tomography- 15 years in cardiology. Circ. J. 77, 1933–1940 (2013). 23856651 10.1253/circj.cj-13-0643.1

[b21] Colchester R. J. . Broadband miniature optical ultrasound probe for high resolution vascular tissue imaging. Biomed. Opt. Express 6, 1502–1511 (2015). 25909031 10.1364/BOE.6.001502 PMC4399686

[b22] Pulido T. . Macitentan and morbidity and mortality in pulmonary arterial hypertension. N. Engl. J. Med. 369, 809–818 (2013). 23984728 10.1056/NEJMoa1213917

[b23] Li N., Zhang S., Hou J., Jang I. K. & Yu B. Assessment of pulmonary artery morphology by optical coherence tomography. Heart Lung Circ. 21, 778–781 (2012). 22884790 10.1016/j.hlc.2012.07.014

[b24] Ishii M. . Evaluation of pulmonary artery histopathologic findings in congenital heart disease: an *in vitro* study using intravascular ultrasound imaging. J. Am. Coll. Cardiol. 26, 272–276 (1995). 7797762 10.1016/0735-1097(95)00154-r

