# Supplementary material for: Optical coherence tomography evaluation of pulmonary arterial vasculopathy in Systemic Sclerosis
Source: Sci Rep. 2017 Feb 24;7:43304. doi: 10.1038/srep43304 (PMC5324107; doi:10.1038/srep43304)
Supplement: Supplementary Information [file srep43304-s1.pdf]

Supplementary data S1

Optical coherence tomography evaluation of pulmonary arterial vasculopathy in Systemic Sclerosis

Johannes P. Schwaiger, MD[1*], Christopher D. Loder[1], David Dobarro, MD[1], Thomas Kaier, MD[1]
Sally Reddecliffe1, Benjamin E. Schreiber, MD1,2, Clive Handler, MD1, Christopher P. Denton, Prof.2, John G. Coghlan, MD1
1 Department of Cardiology, Royal Free London NHS Foundation Trust, Pond Street, London, NW3 2QG, United Kingdom
2 Department of Rheumatology. Royal Free London NHS Foundation Trust, Pond Street, London, NW3 2QG, United Kingdom

	pull-back ID	position	Vessel diameter	IMTA
Patient 1	03:39:48	LL	2,0	33
	03:43:07	LL	2,0	30
	03:48:44	RL	2,2	35
Patient 2	02:57:33	RL	2,0	27
	03:00:22	RL	1,9	34
Patient 3	02:11:59	LL	1,8	27
	02:11:59	RL	2,0	27
Patient 4	03:16:15	RL	2,1	27
	03:22:29	RL	2,2	26
	03:31:36	RU	1,9	35
Patient 5	04:31:08	LL	1,8	30
	04:31:08	LL	1,8	30
Patient 6	05:10:05	RL	2,1	20
	05:18:08	RL	2,2	28
Patient 7	03:50:45	LL	2,0	38
	03:50:45	LL	1,8	38
Patient 8	12:32:12	RL	1,8	36
	12:38:09	RL	1,8	36
	12:44:07	LL	2,0	30
Patient 9	03:11:43	RL	1,9	20
	03:15:42	LL	2,2	28
Patient 10	03:11:45	RL	1,9	26
	03:18:42	RU	2,2	23
	03:23:27	LL	2,3	26
Patient 11	04:23:27	RL	2,0	25
	04:23:27	RL	1,8	22
Patient 12	03:57:21	LL	2,1	28
	04:08:05	RL	2,0	34
Patient 13	04:11:00	RL	2,1	19
	04:15:10	LL	2,2	13
Patient 14	10:26:13	LL	2,0	23
	10:29:50	RL	2,0	24
Patient 15	04:46:03	RL	2,2	30
	04:49:13	RL	2,1	28
	05:04:41	LL	2,1	27
Patient 16	11:41:27	RL	2,0	17
	12:04:38	LL	2,2	16
Control 1	03:51:39	LL	1,8	21
	03:51:39	LL	2,2	21
Control 2	05:36:06	RL	2,2	21
	05:39:52	RL	2,2	24

	05:44:43	RL	1,9	22
Control 3	11:38:04	RL	2,2	21
	11:41:43	RL	1,9	19
Control 4	03:01:55	LL	2,0	17
	03:05:59	LL	2,2	26
Control 5	03:20:49	RL	2,1	21
	03:31:27	RU	1,9	19
	03:33:37	LL	2,2	16

RL	right lower lobe
LL	left lower lobe
RU	right upper lobe
